# Vitamin D Supplementation Enhances Cognitive Outcomes in Physically Active Vitamin D-Deficient University Students in the United Arab Emirates: A 10-Week Intervention Study

**DOI:** 10.3390/nu17172869

**Published:** 2025-09-04

**Authors:** Sarah Dalibalta, Reem Khalil, Rami Baghdan, Sylvie Sekian, Gareth W. Davison

**Affiliations:** 1 Department of Biology, Chemistry and Environmental Sciences, American University of Sharjah, Sharjah P.O. Box 26666, United Arab Emirates; rkhalil@aus.edu (R.K.);; 2 Sport and Exercise Science Research Institute, Ulster University, Belfast Campus, York Street, Belfast BT15 1ED, UK; gw.davison@ulster.ac.uk

**Keywords:** vitamin D, physical activity, cognitive performance, United Arab Emirates, intervention

## Abstract

**Background/Objectives:** Vitamin D deficiency is a global epidemic. In certain populations, such as the United Arab Emirates (UAE), low nutritional intake of vitamin D, inadequate exposure to sunlight, and cultural dress codes can lead to deficiencies in blood vitamin D levels, predisposing them to musculoskeletal disorders, diabetes, and cardiovascular diseases. There are also notable associations between vitamin D deficiency, physical inactivity, and lower cognitive performance. The aim of this study was to determine how vitamin D status may affect physical inactivity and cognitive performance in a young UAE population. **Methods:** Primary data were obtained on vitamin D status, cardiorespiratory fitness, body composition, and blood profiles of students in the UAE. Following initial assessment, a cohort of vitamin D-deficient/insufficient individuals participated in a 10-week physical activity intervention (Group A), whilst another cohort was supplemented with 5000 IU vitamin D3 daily and an exercise intervention (Group B). Both groups underwent physiological and biochemical profiling, and the effects of vitamin D supplementation on cognitive function were assessed. Statistical analysis included paired samples *t*-tests between pre- and post-intervention values and the Wilcoxon signed rank test for within-group comparisons and the Mann–Whitney U test for between-group comparisons. **Results:** The findings suggest that physical exercise alone improves overall cardiorespiratory fitness, as shown by an increased VO_2_ max (*p* < 0.05), while vitamin D supplementation combined with physical exercise did not significantly improve fitness over a 10-week period (*p* > 0.05). However, vitamin D combined with physical exercise significantly improved cognitive performance in Group B only, specifically in working memory, verbal memory, and cognitive flexibility (*p* < 0.05). **Conclusions:** This study highlights the need for targeted interventions such as physical exercise and vitamin D supplementation to be conducted at an early stage in order to improve physical and cognitive function and reduce the risk of disease.

## 1. Introduction

Vitamin D (calciferol) is a fat-soluble vitamin with hormonal functions, obtained from the diet or by supplementation, although it is largely synthesized in the skin from cholesterol by ultraviolet sunlight exposure [1]. The final active product, calcitriol (1.25 dihydroxyvitamin D), circulates in the bloodstream, regulating calcium metabolism and bone mineralization and promoting healthy bones and growth [1]. This vitamin has also been found to affect neuromuscular and immune function, and potentially plays an important role in the progression of chronic diseases such as diabetes, cardiovascular disease (CVD), and many types of cancer [2]. Whilst CVD and diabetes are stand-alone diseases, there is a close association between vitamin D deficiency, physical inactivity, and increased risk of these disease types. In healthy adults, vitamin D status has an inverse relationship with the risk of type 2 diabetes and metabolic syndrome; therefore, maintaining a sufficient blood concentration of vitamin D may be a useful preventative measure for metabolic diseases [3].

The exact mechanism by which vitamin D deficiency reduces mortality from CVD is yet unknown. Nonetheless, vitamin D is thought to down-regulate pro-inflammatory markers and up-regulate anti-inflammatory factors, thereby reducing the risk of atherogenesis [4]. Higher vitamin D concentrations have also been shown to modify the blood lipid profile, potentially decreasing the incidence of CVD [5]. Physical inactivity is inextricably linked to the onset of CVD and diabetes, and it has also been postulated that vitamin D deficiency is linked to exercise intolerance and physical inactivity [6]. Vitamin D deficiency is also prevalent in athletes, with a significantly lower vitamin D status in indoor compared to outdoor athletes [7]. To improve physical performance, athletes have been previously subjected to vitamin D-producing ultraviolet irradiation, which showed improvements in speed, cycling performance, cardiovascular fitness, and muscular endurance [8]. Athletes also seem to perform better in the summer months than in the winter months [9]. Muscle biopsy studies in humans confirm that treatment with vitamin D increases protein synthesis, muscle hypertrophy, and strength, and muscle regeneration after injury [9].

The presence of vitamin D receptors (VDRs) in the brain has fueled research assessing the relationship between vitamin D and brain function. For example, there is an association between vitamin D and diseases such as Alzheimer’s and dementia, and a 4-fold risk of cognitive decline and dementia in vitamin D-deficient elders compared to peers with adequate vitamin D [10]. Hypovitaminosis D is understood to expose neurons to aging and Alzheimer’s type neurodegeneration [11], with higher vitamin D in the brain being associated with better cognitive function prior to death [12]. On the other hand, a randomized controlled trial of 63 young adults (18–30 years old) supplemented with vitamin D for six weeks showed no significant changes in working memory, response inhibition, and cognitive flexibility [13]. Although this area of research is still preliminary, vitamin D status and cognitive function appear to be highly related.

Vitamin D deficiency is a global health issue, with deficiencies highly prevalent in the Eastern Mediterranean region and lower-middle-income countries [14]. Serum 25-hydroxyvitamin D (25-OHD) is used to determine vitamin D status. Vitamin D deficiency in the UAE in various age groups remains alarmingly high, with 82.5% of the population deemed deficient [15]. In general, serum 25-OHD appears to be lower at higher altitudes; in darker skin types; in individuals who stay out of the sunshine, wear skin-covering clothes, and use high-factor sun block; and particularly in women. In the UAE, women of childbearing age have considerably low concentrations of serum 25-OHD (average of 8.6 ng/mL or 21.5 nmol/L), mainly due to limited sun exposure [16]. Another study on Emirati women also indicated a high frequency of vitamin D deficiency, as all 259 women tested were found to be vitamin D-deficient, with a mean serum 25-OHD concentration of about 25 nmol/L [17]. Moreover, Arab infants and children in the UAE have been shown to have poor vitamin D values; 22% with serum 25-OHD < 25 nmol/L [18]. A study on young university students in Abu Dhabi (n = 208) showed that seasonal variation played an important role in the vitamin D status of individuals in the UAE [19]. Nonetheless, vitamin D deficiency appears to be prevalent in this young population and was validated further by another study conducted in Al-Ain, UAE [20]. This study, conducted on adolescents (n = 315) aged 15–18 years old, showed that 65% of participants were either vitamin D-deficient or insufficient, with girls exhibiting a greater prevalence than boys. Interestingly, this work also reported an inverse relationship between 25-OHD concentrations and BMI, and being positively and significantly correlated with physical activity scores. Another study of undergraduate female UAE college students showed that almost 50% had suboptimal serum vitamin D levels [21]. Moreover, the UAE has the second-highest prevalence of type 2 diabetes in the world, and CVD accounted for more than 25% of all deaths in the UAE in 2010 [22].

These statistics highlight that public health measures are required to assess and correct the vitamin D status of the UAE population. A central goal of this study was, therefore, to test the effectiveness of a combined approach of vitamin D supplementation and physical activity on physiological and biochemical measures in a sample of young UAE adults. Secondly, this study aimed to assess cognitive function in individuals deficient in vitamin D compared to those with sufficient vitamin D levels after 10 weeks of supplementation and a physical exercise intervention.

## 2. Materials and Methods

### 2.1. Participant Selection

Upon ethical approval and written informed consent, 32 ethnically diverse students (24 females and 8 males) at the American University of Sharjah (AUS) participated in this study in the Fall term (months of September-December). Participants were recruited through university-wide announcements and email invitations. The inclusion criteria required students to be aged 18–23 years old and physically able to participate in moderate–high-intensity exercise of their choice over a 10-week period. Participants would be free from any chronic illness or musculoskeletal conditions that would limit exercise participation and would be willing to complete a minimum of 3 h of moderate to high-intensity physical exercise every week as recommended by the World Health Organization (WHO) for a total of 10 weeks. There was no limit on gender or minority status. Low vitamin D status (serum 25-OHD < 30 ng/mL) was an inclusion criterion for participation in the supplementation intervention group. The exclusion criteria included current vitamin D supplementation, recent fractures, or injuries. The participants were screened for this study using the above inclusion criteria. Randomization was carried out after baseline vitamin D levels were assessed using a computer-generated simple randomization method. The participants were allocated into two groups reflecting natural variability in gender distribution; Group A (n = 13) comprised 7 males and 6 females, and Group B (n = 19), comprising 2 males and 17 females. The study cohort included individuals of Arab-Emirati and non-Emirati ethnic backgrounds and participants of African, Asian, European, and American origin. The average participant age in our cohort was 21 years old (M = 21 and SD = ±1.2).

### 2.2. Research Protocol

To assess the participants’ cardiorespiratory fitness, various physiological and biochemical measurements were taken before the start of the exercise intervention. Physiological parameters included height and weight, and waist and hip circumference, presented as a ratio. A waist/hip ratio correlates significantly with cardiovascular risk—the lower the ratio, the lower the cardiovascular risk. In females, a ratio of 0.85 or lower, and in males, a ratio of 0.9 or lower was considered healthy. Other measurements included body mass index (BMI), blood pressure (diastolic and systolic), and pulse rate with the use of a sphygmomanometer (Pulssecor Cardioscope BP+ Uscom, Hampshire, UK), and muscle mass, fat mass, and fat percentage measured using a Body Composition Analyzer (BCA-Tanita). The maximum oxygen volume (VO_2_ max) was measured using a running beep test to assess cardiorespiratory fitness.

The biochemical parameters included analysis of fasting blood vitamin D3, triglycerides, and cholesterol (total, HDL, LDL, and HDL/total ratio) conducted at the AUS health clinic. Following these measurements, the participants were grouped randomly into two groups after vitamin D3 levels were assessed. Serum 25-hydroxyvitamin D (25-OHD) was used to determine vitamin D status. Vitamin D deficiency is defined as having serum 25-OHD < 20 ng/mL (50 nmol/L), while vitamin D insufficiency is recognized as 25-OHD between 21 and 29 ng/mL (50–75 nmol/L). Optimal levels of vitamin D are 30–50 ng/mL (75–125 nmol/L) [23]. Group A (n = 13) were participants who had vitamin D deficiency/insufficiency but were not supplemented. Group B (n = 19) were participants who were vitamin D-deficient/insufficient and were supplemented with one tablet of 5000 IU Vitamin D3 (Solgar, Lakewood, NJ, USA) daily. All the participants took part in a 10-week exercise intervention program, involving moderate–high-intensity exercise (such as brisk walking, running, cycling, swimming, etc.) for at least three hours a week. To ensure the participants were following the program, exercise diaries were kept by each individual explaining their type and duration of exercise, and were checked on a weekly basis. After the 10 weeks, the measurements were repeated. In addition, the participants (n = 29) took part in a battery of cognitive tests both at the start and the end of the intervention to measure their performance in executive function, planning, working memory, visual memory, new learning, episodic memory, visual recognition memory, spatial working memory, and verbal recognition memory.

### 2.3. CANTAB Software

Cognitive assessments are important tools for understanding the role of specific brain functions. The Cambridge Neuropsychological Test Automated Battery (CANTAB v1.6) includes highly sensitive and objective measures of cognitive function. The tests are used to detect changes in neuropsychological performance and include tests of working memory; learning executive function; visual, verbal, and episodic memory; attention, information processing, and reaction time; social and emotional recognition; decision making; and response control “cambridgecognition.com (accessed on 25 May 2025)”. The battery of tests used for the purposes of this experiment included One Touch Stocking of Cambridge (OTS), Paired Associates Learning (PAL), Pattern Recognition Memory (PRM), spatial working memory (SWM), and verbal recognition memory (VRM).

One Touch Stocking of Cambridge (OTS) tests each participant’s executive function, planning, and working memory based upon the “Tower of Hanoi”. Participants see two displays containing three colored balls and must work out in their head how many moves would be required to make the lower display match the upper display. In this test, latency and accuracy measures were calculated.

The Paired Associates Learning (PAL) test assesses participants’ visual memory and new learning. It is a sensitive tool for accurate evaluation of episodic memory. In this test, boxes are displayed on the screen and open one by one in a random order to reveal patterns hidden inside. Afterwards, the patterns are displayed in the center of the screen, one at a time, and the participant must touch the box where the pattern was originally located. If the participant makes an error, the patterns are presented once again to remind the participant of their locations.

The Pattern Recognition Memory (PRM) test measures visual recognition memory. Participants watch a series of 12 patterns appear, one at a time, on the screen. These patterns are designed so that they cannot be given verbal labels. In the recognition phase of the test, the participant chooses which two patterns they have already seen before. Afterwards, this is repeated with a new set of 24 patterns to be remembered.

The spatial working memory (SWM) test requires retention and manipulation of visuospatial information. This test has executive function demands and measures strategy use and errors. The test begins with colored boxes being shown on the screen. The aim of the test is that, by touching the boxes and using a process of elimination, the participant should find one token in each of the boxes and use them to fill up an empty column on the right-hand side of the screen. The key task instruction is that the computer will never hide a token in the same-colored box, so once a token is found in a box, the participant should not return to that box to look for another token. The color and position of the boxes used are changed from trial to trial to discourage the use of stereotyped search strategies. The key outcome measures for SWM include errors (touching boxes that have been found to be empty and revisiting boxes that have already been found to contain a token) and strategy, a measurement of executive function.

The verbal recognition memory (VRM) test measures the ability to encode and subsequently retrieve verbal information. This test contains 3 elements: a presentation phase, a free recall phase, and a recognition phase. The participant is initially shown a list of words that they are required to remember. If a free recall phase is included, the participant must then say the words they remember seeing while the experimenter logs what words are spoken. A forced-choice recognition phase can then be selected, asking the participant to say whether they remember seeing the word on screen before. The word can be one of the originals, or a new word (distractor) that they have not seen before.

Each test has a few key outcome measures that are especially important. These are OTSMDLFC and OTSPSFC for the OTS test; PALFAMS28 and PALTEA28 for the PAL test; PRMPCD and PRMPCI for the PRM test; SWMBE12, SWMBE4, SWMBE468, SWMBE6, SWMBE8, and SWMS for the SWM test; and VRMDRTC, VRMFRDS, and VRMIRTC for the VRM test. Table 1 describes each of the key outcome measures.

### 2.4. Statistical Analysis

A paired samples *t*-test was conducted between all the pre- and post-intervention values for biochemical, physiological, and cognitive parameters. Statistical significance set at a *p* ≤ 0.05. Given that our cognitive data did not conform to the assumptions of normality, we employed two primary non-parametric tests: the Wilcoxon signed rank test for within-group comparisons and the Mann–Whitney U test for between-group comparisons. This approach is especially useful in the context of our study, where the sample sizes are small and not normally distributed. An a priori power analysis was performed (G*Power V 3.1.9.7) based on data from Petterson et al., 2017 [24], where a significant difference in cognitive function was observed following vitamin D supplementation. Assuming α = 0.05, power = 0.80, and effect size = 0.75 (moderate), it was estimated that 24 participants would be necessary to detect differences in cognitive function with Vitamin D supplementation. A significant result from the Wilcoxon signed rank test indicates that supplementation had a statistically significant effect on the cognitive outcomes within the group, suggesting that the changes observed are unlikely to have occurred by chance. The Mann–Whitney U test was used to evaluate the differences in change scores between the two independent groups (non-supplemented and supplemented). To control for Type I error due to multiple comparisons in the cognitive data, the Bonferroni correction was applied to all between-group analyses. The corrected significance threshold was set at α = 0.0033 based on 15 comparisons.

## 3. Results

### 3.1. Physiological Results

Cardiorespiratory fitness was assessed through conducting physiological and biochemical measurements. All the volunteer participants were vitamin D-deficient/insufficient and then randomly divided into Group A (n = 13) or Group B (n = 19). Table 2 shows the mean physiological parameters for the participant cohort pre and post the exercise intervention. After statistical analysis, the VO_2_ max value was the only physiological parameter that was different within each group, pre and post the exercise intervention. No physiological differences were observed between Groups A and B.

Similarly, the mean and standard deviations of the biochemical parameters were calculated for the entire cohort as shown in Table 3 both pre and post the exercise intervention. From the biochemical parameters, cholesterol, triglycerides, and LDL-cholesterol values were found to be significantly different pre and post the intervention in Group A. That small increase observed may be attributed to dietary changes that were not controlled for in this study. Group A participants also had a mean 25-OHD concentration of 22 ± 3 ng/mL at baseline compared to Group B participants, who had a mean 25-OHD concentration of 13 ± 4 ng/mL at baseline. Unsurprisingly, vitamin D values were found to be significantly higher in the supplemented Group B (32 ± 9 ng/mL) post-intervention compared to the mean 25-OHD concentration of 23 ± 6 ng/mL in Group A. No other biochemical parameters were found to be significantly different between Groups A and B.

### 3.2. Cognitive Results

A battery of cognitive tests was administered before and after a 10-week intervention to assess the effects of vitamin D on cognitive performance. These tests measured executive function, working memory, and visual and verbal memory. Group A (vitamin D-deficient/insufficient and not supplemented) and Group B (vitamin D-deficient/insufficient and supplemented) participants were required to exercise for 3 h every week in a moderate-vigorous range for 10 weeks, and the same battery of tests was conducted after the 10-week intervention. A paired *t*-test was performed to check for significant differences between pre-intervention and post-intervention values for all the participants; however, the difference between values was considered to be not statistically significant.

Table 4 compares the results of the key outcome measures between groups using the Mann–Whitney U test to determine whether groups differed in change scores (post minus pre values). The table also shows the results from the Wilcoxon signed-rank test, which analyzed differences from pre to post within each group. Although both groups, Group A (non-supplemented) and Group B (vitamin D-supplemented), participated in the same physical exercise protocol, only the participants in the supplemented group improved across several cognitive domains. For example, in the OTS task, which assesses planning and executive function, Group B had faster response times and greater accuracy, with significant improvements in both latency (OTSMDLFC; *p* = 0.01) and first-choice problem solving (OTSPSFC; *p* = 0.03). However, these measures did not remain statistically significant after Bonferroni correction (OTSMDLFC; *p* = 0.15) and (OTSPSFC; *p* = 0.45). Moreover, spatial working memory also improved in the supplemented group, with fewer errors (SWMBE4; *p* = 0.02) and more efficient strategies (SWMS; *p* = 0.04). While these measures showed a potentially meaningful trend, they did not meet the Bonferroni-adjusted criterion and should be interpreted with caution (SWMBE4; *p* = 0.3) and (SWMS; *p* = 0.06). Likewise, the supplemented group also showed significant improvements in the verbal memory outcomes, such as immediate recognition (VRMIRTC; *p* = 0.001) and free recall (VRMFRDS; *p* = 0.03). However, only in the immediate recognition task did this change remain significant after a Bonferroni correction (VRMIRTC; *p* = 0.015). These findings suggest that after accounting for multiple comparisons, most observed group differences were not statistically significant. The findings based on uncorrected *p*-values should, therefore, be interpreted with caution.

## 4. Discussion

Although the Middle East enjoys ample amounts of sunshine, numerous issues, such as religious practices, cultural dress codes, limited exposure to sunlight, darker skin color, and an inadequate dietary intake of vitamin D, can all lead to vitamin D deficiency. In the UAE, 90% of the population is understood to be vitamin D-deficient [27]. This can have notable health consequences, including bone disease, muscle weakness, cancer, and immune malfunction. In parallel to vitamin D deficiency, the UAE also has a high prevalence of CVD and diabetes, whilst a large proportion of the population is known to be physically inactive [28,29,30]. According to the WHO, adults should engage in at least 150 min of moderate-intensity or 75 min of high-intensity physical activity per week [31]. The Dubai Household Health Survey conducted in 2009 found that only 19% of people in Dubai exercised regularly to maintain a healthy lifestyle [32]. Children and adolescents can increase their vitamin D levels by being physically active outdoors and getting more exposure to the sun, as exercise in itself may contribute to the maintenance of vitamin D status [33]. This correlation could be affiliated with increased metabolic clearance and enhanced uptake of vitamin D in adipose tissue, accounting for the low levels of 25-OHD serum in overweight and obese individuals [34]. Vitamin D deficiency in this population may, therefore, be a contributing factor and could be linked to the rise of physical inactivity and CVD. Hence, vitamin D deficiency may present as a limiting factor when describing physical activity, muscle strength, and performance. Moreover, vitamin D levels are thought to be correlated with cognitive performance in older adults [35,36,37], yet the relationship between blood vitamin D and cognitive function in young adults has not been thoroughly explored. To this end, the central goal of this study was to test the additive effects of vitamin D supplementation and exercise intensity on cardiorespiratory fitness and cognitive performance in a cohort of students.

Our findings suggest that physical exercise alone, as shown in Group A, improves overall cardiorespiratory fitness as determined by an increase in VO_2_ max. However, vitamin D supplementation combined with physical exercise, as shown in Group B, did not significantly improve fitness. This was similar to a study that found moderate doses of vitamin D supplementation did not have a significant improvement in the physical performance, exercise capacity, or physical activity of participants [38]. However, when 25-OHD concentrations reached 60 nmol/l after intervention, a borderline significant improvement was observed in exercise capacity [38]. In other words, there may be a relationship between vitamin D concentrations and physical activity. A study on a group of 6–17-year-old vitamin D-deficient Saudi children showed positive correlations with volume of physical activity, as 25-OHD levels were lowest in children who were the least physically active [39]. Thus, it could be speculated that as vitamin D deficiency increases in the UAE population, physical activity would decrease, and as a result, CVD cases may increase. In fact, in an obese Emirati population of type 2 diabetics, there was a negative correlation between serum 25-OHD and LDL-cholesterol, total cholesterol, triglycerides, BMI, and waist circumference [40]. A recent study on Arab adolescent boys, aged 13–17 years, demonstrated that 25-OHD had an inverse relationship with BMI, blood pressure, glucose, and triglycerides, and a positive association with HDL-cholesterol. In girls, however, 25-OHD was only inversely associated with hip circumference and skin color and positively associated with HDL-cholesterol [41]. Therefore, appropriate levels of serum vitamin D play an important role in preventing or controlling diabetes and improving cardiovascular function.

We also assessed cognitive performance after 10 weeks of vitamin D3 supplementation and a physical exercise program, and showed that in Group B, vitamin D supplementation combined with physical exercise significantly improves cognitive performance, whereas in Group A, physical activity alone leaves cognitive performance unaltered. In the supplemented group, we observed an increase in the number of problems solved on the first choice and a decrease in mean latency to correct response, suggesting that working memory and cognitive flexibility may have improved. Likewise, participants in the supplemented group made fewer errors in the 4-token condition and outperformed participants with a higher strategy. This suggests a potential protective effect of vitamin D on working memory under high cognitive load. Of note, the above-mentioned measures did not remain significant after correcting for multiple comparisons. However, significant cognitive enhancements were observed in the verbal memory performance, such as in verbal recognition memory, even after applying a Bonferroni correction (VRMIRTC; *p* = 0.015), further implicating vitamin D’s role in supporting long-term memory consolidation. Although there is robust evidence that suggests physical activity alone does indeed improve cognitive function [42,43], we did not observe this in the non-supplemented group. Meta-analysis of adults older than 50 years old found that exercise improved cognition with combined moderate-intensity aerobic and resistance training [44]. Additionally, Erickson et al. (2011) showed that in sedentary older adults, a 12-month aerobic exercise trial increased anterior hippocampal volume corresponding to gains in spatial memory [45]. We did not observe these changes in our study, potentially due to the small sample size. However, our findings align with the literature supporting vitamin D’s neuroprotective role in cognitive function. Specifically, vitamin D enhances neuroplasticity through genomic and non-genomic pathways that converge on neurogenesis, synaptogenesis, and long-term potentiation (LTP) [46]. Genomically, vitamin D up-regulates neurotrophins such as BDNF, NGF, NT-3, and GDNF, and their receptors (TrkA, TrkB, p75NTR), thereby promoting neuronal differentiation, maturation, and survival [47,48,49]. It also enhances the expression of synaptic proteins like CaMKII, GluA1, synapsin, and PSD-95, which are important effectors in synaptic consolidation and structural plasticity [50,51]. Non-genomically, vitamin D activates calcium signaling via L-type voltage-gated calcium channels and Wnt/CaMKII pathways, contributing to dendritic remodeling and synaptic formation [52]. Animal studies further demonstrate that vitamin D supplementation enhances hippocampal LTP, a key process in synaptic strengthening and memory formation [53]. Vitamin D supplementation also leads to increases in neuronal excitability and facilitates glutamatergic neurotransmission, which are fundamental to learning and memory processes [54]. Together, these mechanisms position vitamin D as a potent modulator of experience-dependent brain plasticity. Very few studies have assessed the effects of vitamin D supplementation on cognitive performance in a young adult cohort. Given that brain regions such as the prefrontal cortex (PFC) undergo protracted maturation well into the third decade of life [55], maintaining adequate vitamin D levels during adolescence and early adulthood is critical to support optimal cognitive development, particularly in at-risk populations. Pettersen (2017) found that high-dose vitamin D supplementation significantly increased serum 25-OHD levels and improved performance on the Pattern Recognition Memory (PRM) and Paired Associates Learning (PAL) tasks in young healthy adults [24]. This was particularly true among participants with baseline insufficiency (<75 nmol/L). These gains in visual memory were not observed in the low-dose group, indicating a dose-dependent benefit on cognitive tasks reliant on nonverbal memory. Another study found that oral vitamin D supplementation over 18 weeks enhanced multiple cognitive domains, including memory, attention, and executive function in healthy adolescent girls [56]. The most pronounced effects were observed in nonverbal (visual) memory, supporting a link between vitamin D status and executive function-dependent cognitive processes. Similarly, a more recent study by Bailey and Pettersen (2024) showed that vitamin D insufficiency was prevalent in adolescents, and higher vitamin D levels were linked to better visual memory, particularly in older participants, suggesting potential age-related effects on cognition [57]. In elderly populations, the observed cognitive benefits of vitamin D supplementation are often attributed to its role in mitigating neurodegeneration, vascular pathology, and inflammation, processes that are more pronounced with age [36,42]. In contrast, the young adult brain is characterized by ongoing synaptic pruning, neuroplasticity, and continued maturation of the frontal cortex [56]. These developmental features may render younger individuals more responsive to interventions that modulate neurotrophic factors and synaptic signaling, especially when combined with physical activity.

There were several limitations in this study. The absence of a ‘pure’ non-exercise, non-supplemented control group may be a drawback, as the effects of vitamin D supplementation cannot be isolated from concurrent physical activity. The sample size was also small, sex-imbalanced, and ethnically diverse but unstratified, limiting our ability to explore demographic or subgroup effects. The dietary habits of these university students were not assessed, although nutrition is known to significantly impact both the physical and mental well-being of individuals. Tokarshuk et al., 2022, highlighted how a 90-day intervention combining nutrition, physical activity, and synbiotics significantly improved vitamin D levels, body composition, and emotional well-being in vitamin D-deficient university students due to a synergistic lifestyle and gut microbiota modulation [58]. Moreover, serum 25-OHD is known to rise gradually with continued supplementation, taking several weeks to reach a plateau [59]. Hence, it is likely that the average serum 25-OHD concentrations are lower during the study period than the eventual peak levels achieved with prolonged supplementation. If a steady state level of serum 25-OHD is required to exert significant effects, then the full potential effects of vitamin D supplementation on physiological or cognitive effects may be limited. Future studies should perhaps investigate longer supplementation periods or higher doses to fully evaluate the effects of vitamin D. Despite that, these results collectively suggest that vitamin D supplementation, when combined with exercise, can potentially improve executive function, spatial working memory, and verbal memory in young adults more effectively than exercise alone. This positive relationship between vitamin D supplementation and physical exercise intervention with cognitive performance needs to be explored further with a greater number of participants in order to draw firm conclusions.

## 5. Conclusions

Vitamin D deficiency is a significant public health issue globally, but particularly in the UAE. A few studies involving Arab populations point to a beneficial association between high vitamin D and low diabetes and CVD risk. Therefore, appropriate levels of serum vitamin D may play an important role in preventing or controlling diabetes and improving cardiovascular function. The very high prevalence of vitamin D deficiency in the UAE and other Arab populations, also motivates cognitive studies in these settings. This study aimed to correct vitamin D deficiency by supplementation and subsequently determine its effectiveness on risk factors associated with physical inactivity and performance on multiple cognitive tests. Preliminary results show a positive impact on cognitive performance, even more than cardiorespiratory fitness, with a combined vitamin D and exercise intervention approach. These results highlight the need for routine lifestyle changes such as physical exercise and vitamin D supplementation to markedly enhance quality of life and improve physical and cognitive function. Future studies should aim to replicate these findings in a larger and more balanced cohort to ensure adequate power for subgroup analyses and enhance external validity and generalizability. Moreover, future studies may increase the duration of the exercise and supplementation intervention to help quantify the magnitude of change associated with regular exercise and increased vitamin D levels of greater than 10 weeks. We also suggest incorporating sleep and dietary tracking and sunlight exposure logs in future iterations of this study to better control for these potential confounders. Other cognitive performance tests may also be investigated to further understand the impact of vitamin D supplementation on cognition. This study however, provides preliminary data for public health authorities and policy makers in the UAE, which will be essential in developing effective intervention strategies for preventing and controlling the long-term risks of vitamin D deficiency in this population.

## Figures and Tables

**Table 1:** Description of CANTAB tests and cognitive functions measured.

Table	Measure Name	Measure Description	Cognitive Function
OTS	OTSMDLFC	KEY: OTS Median Latency to First Choice: The median latency, measured from the appearance of the stocking balls until the first box choice was made by the subject. Calculated across all assessed trials where the subject’s first response was correct.	Spatial planning; spatial working memory; executive function
OTS	OTSPSFC	KEY: OTS Problems Solved on First Choice: The total number of assessed trials where the subject chose the correct answer on their first attempt. Calculated across all assessed trials.	0
PAL	PALFAMS28	KEY: PAL First Attempt Memory Score: The number of times a subject chose the correct box on their first attempt when recalling the pattern locations. Calculated across assessed trials, omitting the 12 box level to provide a direct comparison to the Recommended Standard	Visual episodic memory; learning
PAL	PALTEA28	KEY: PAL Total Errors (Adjusted): The number of times the subject chose the incorrect box for a stimulus on assessment problems (PALTE), plus an adjustment for the estimated number of errors they would have made on any problems, attempts, and recalls they did not reach. This measure allows you to compare performance on errors made across all subjects, regardless of those who terminated early versus those who completed the final stage of the task. In this task variant, PALTEA does not include the 12 box level to provide a direct comparison to the Recommended Standard.	0
SWM	SWMBE4	KEY: SWM Between errors 4 boxes: The number of times a subject revisits a box in which a token has previously been found. Calculated across all trials with 4 tokens only.	0
SWM	SWMS	KEY: SWM Strategy (6–8 boxes): The number of times a subject begins a new search pattern from the same box they started with previously. If they always begin a search from the same starting point, we infer that the subject is employing a planned strategy for finding the tokens. Therefore, a low score indicates high strategy use (1 = they always begin the search from the same box), a high score indicates that they are beginning their searches from many different boxes. Calculated across assessed trials with 6 tokens or 8 tokens.	Spatial memory; spatial working memory; heuristic strategy; executive function
VRM	VRMFRDS	KEY: VRM Free Recall: Distinct Stimuli: The total number of distinct words that are correctly recalled from the presentation phase by the subject during the immediate free recall stage.	Verbal memory
VRM	VRMIRTC	KEY: VRM Immediate Recognition: Total Correct: The total number of target words that the subject correctly recognizes, plus the total number of distractor words that the subject correctly rejects.	0

**Table 2:** Physiological and fitness indices of Groups A and B at baseline and following 10 weeks of a physical activity intervention. † denotes a main effect for time (pre vs. post) within a group (*p* ≤ 0.05).

	Physiological Parameters			
n = (32)	Group A n = (13)		Group B n = (19)	
	Vitamin D insufficient/not supplemented		Vitamin D insufficient/supplemented	
Mean Values ± SD	Pre	Post	Pre	Post
BMI (kg/m^2^)	25 ± 8	24 ± 8	25 ± 6	24 ± 5
VO_2_ Max (mL/kg/min)	29 ± 6	32 ± 8 (†)	27 ± 4	31 ± 4 (†)
Waist/Hip Ratio	0.83 ± 0.10	0.82 ± 0.20	0.80 ± 0.10	0.83 ± 0.10
Systolic Blood Pressure (mmHg)	123 ± 22	113 ± 16	115 ± 15	114 ± 10
Diastolic Blood Pressure (mmHg)	74 ± 9	70 ± 10	67 ± 10	67 ± 8
Pulse Rate (bpm)	82 ± 12	83 ± 21	76 ± 10	81 ± 11
Fat Percentage (%)	26 ± 11	26 ± 10	32 ± 9	31 ± 9
Fat mass (kg)	20 ± 20	20 ± 14	22 ± 10	21 ± 10
Muscle mass (kg)	50 ± 17	49 ± 17	43 ± 10	43 ± 10

**Table 3:** Biochemical indices of Groups A and B at baseline and following 10 weeks of a physical activity intervention. † denotes a main effect for time (pre vs. post) within a group (*p* ≤ 0.05); * denotes a main effect for group (Group A vs. B) (*p* ≤ 0.05).

			Biochemical Parameters		
n = (32)	Group A n = (13)		Group B n = (19)		
	Vitamin D insufficient/not supplemented		Vitamin D insufficient/supplemented		Reference Ranges [23,25,26]
Mean Values ± SD	Pre	Post	Pre	Post	
Cholesterol (mg/dL)	166 ± 27	171 ± 17 (†)	180 ± 30	180 ± 30	Desirable: <200 Borderline: 200–239 High: > or =240
Triglycerides (mg/dL)	70 ± 30	90 ± 40 (†)	61 ± 15	63 ± 15	Normal: <150 High: 200–499 Very High: > or =500
LDL-cholesterol (mg/dL)	95 ± 23	106 ± 20 (†)	107 ± 21	112 ± 26	Optimal: <100 Near or above optimal: 100–129 Borderline High: 130–159 High: 160–189 Very High: > or =190
HDL-cholesterol (mg/dL)	53 ± 14	54 ± 12	56 ± 13	57 ± 11	Heart Disease Risk Major Risk: <40 Negative Risk: > or =60
Total cholesterol/HDL-cholesterol Ratio	3.3 ± 0.7	3.2 ± 0.7	3.2 ±0. 6	3.2 ± 0.7	Ideal: Below 3.5 Acceptable: 4.5–5.0 High Risk: Above 5.0 Very High Risk: Above 6.0
Vitamin D3 25-OH (ng/mL) (*)	22 ± 3	23 ± 6	13 ± 4	32 ± 9 (†)	Deficiency: <20 Insufficiency: 21–29 Sufficiency: 30–50 Excess: 50–100 Toxicity: >150

**Table 4:** Results of the key outcome measures within each group using the Wilcoxon signed rank test and between groups using the Mann–Whitney U tests. Statistically significant differences are indicated in BOLD = *p* < 0.05.

	Group A (n = 10)	Group B (n = 19)	Between-Group Change Score Comparison
	Median (Range)	Median (Range)	Mann–Whitney U Test
SWMBE6			U = 98.0, *p* = 0.93, r = 0.00
Pre-intervention	1 (0–6)	0.83 (0–8)	
Post-intervention	1.14 (0–11)	0.5 (0–10)	
Wilcoxon signed rank test	(Z = −0.53, *p* = 0.72)	(Z = −0.1, *p* = 0.93)	
OTSMDLFC			U = 94.0, *p* = 0 .81, r = 0.00
Pre-intervention	10,700 (5700–28,600)	11,200 (5282–21,131)	
Post-intervention	10,900 (6201–15,377)	8000 (4189–15,725)	
Wilcoxon signed rank test	(Z = −1.38, *p* = 0.19)	(Z = −2.58, ***p* = 0.01**)	
OTSPSFC			U = 93.0, *p* = 0.77, r = 0.00
Pre-intervention	12 (3–14)	11 (1–14)	
Post-intervention	13 (10–15)	13 (7–15)	
Wilcoxon signed rank test	(Z = −1.97, *p* = 0.06)	(Z = −2.14, ***p* = 0.03**)	
PALFAMS28			U = 52.5, ***p* = 0 .03**, r = 0.15
Pre-intervention	15 (9–19)	17 (9–20)	
Post-intervention	17 (14–20)	18 (14–20)	
Wilcoxon signed rank test	(Z = −2.83, *p* = 0.002)	(Z = −1.38, *p* = 0.18)	
PALTEA28			U = 62.0, *p* = 0 .10, r = 0.10
Pre-intervention	5.8 (1–15)	2.8 (0–18)	
Post-intervention	1.1 (0–4)	1.2 (0–21)	
Wilcoxon signed rank test	(Z = −2.67, ***p* = 0.004**)	(Z = −1.5, *p* = 0.14)	
PRMPCD			U = 88.0, *p* = 0 .60, r = 0.01
Pre-intervention	92 (67–100)	97 (83–100)	
Post-intervention	92 (50–100)	97 (58–100)	
Wilcoxon signed rank test	(Z = −0.09, *p* = 1.00)	(Z = −0.68, *p* = 0.53)	
PRMPCI			U = 66.0, *p* = 0.08, r = 0.10
Pre-intervention	96 (92–100)	99 (92–100)	
Post-intervention	98 (83–100)	99 (50–100)	
Wilcoxon signed rank test	(Z = −1.0, *p* = 0.53)	(Z = −0.65, *p* = 0.66)	
SWMBE12			U = 84.0, *p* = 0 .49, r =0.02
Pre-intervention	30 (12–45)	18 (0–49)	
Post-intervention	20 (7–47)	18 (0–48)	
Wilcoxon signed rank test	(Z = −0.77, *p* = 0.49)	(Z = −0.02, *p* = 0.99)	
SWMBE4			U = 64.5, *p* = 0.07, r = 0.12
Pre-intervention	0.4 (0–2)	0.4 (0–4)	
Post-intervention	0.4 (0–3)	0.1 (0–2)	
Wilcoxon signed rank test	(Z = 0.0, *p* = 1.00)	(Z = −2.53, ***p* = 0.02**)	
SWMBE468			U = 91.0, *p* = 0 .71, r = 0.01
Pre-intervention	11 (0–26)	4.6 (0–25)	
Post-intervention	4 (0–29)	3.5 (0–29)	
Wilcoxon signed rank test	(Z = −1.3, *p* = 0.22)	(Z = −0.94, *p* = 0.36)	
SWMBE8			U = 82.0, *p* = 0.44, r = 0.02
Pre-intervention	10 (0–18)	3 (0–19)	
Post-intervention	4 (0–15)	3 (0–24)	
Wilcoxon signed rank test	(Z = −1.61, *p* = 0.12)	(Z = −0.51, *p* = 0.63)	
SWMS			U = 63.5, *p* = 0.11, r = 0.10
Pre-intervention	8 (2–11)	7 (2–11)	
Post-intervention	8 (2–10)	6 (2–10)	
Wilcoxon signed rank test	(Z = −0.1, *p* = 1.00)	(Z = −2.07, ***p* = 0.04**)	
VRMDRTC			U = 91.5, *p* = 0.72, r = 0.00
Pre-intervention	34 (24–36)	32 (28–36)	
Post-intervention	34 (30–36)	33 (29–36)	
Wilcoxon signed rank test	(Z = −0.57, *p* = 0.66)	(Z = −1.2, *p* = 0.26)	
VRMFRDS			U = 75.0, *p* = 0.28, r = 0.04
Pre-intervention	6.3 (5–12)	6.3 (0–13)	
Post-intervention	7.2 (5–13)	9 (5–15)	
Wilcoxon signed rank test	(Z = −0.86, *p* = 0.52)	(Z = −2.09, ***p* = 0.03**)	
VRMIRTC			U = 86.0, *p* = 0.54, r = 0.01
Pre-intervention	32 (26–35)	30 (26–36)	
Post-intervention	34 (30–35)	33 (29–36)	
Wilcoxon signed rank test	(Z = −1.67, ***p* = 0.01**)	(Z = −3.061, ***p* = 0.001**)	

OTSMDLFC = mean choices to correct for problems with defined difficulty, OTSPSFC = probability of success on the first choice, PALFAMS28 = first trial memory score (total correct responses on the first trial for 2–8 pattern stages), PALTEA28 = total number of errors adjusted for difficulty, PRMPCD = percentage of correct responses during the delayed recognition phase, PRMPCI = percentage correct during immediate recognition, SWMBE4 = between errors on the 4-box trials, SWMBE6 = between errors on the 6-box trials, SWMBE8 = between errors on the 8-box trials, SWMBE468 = aggregates these error scores across 4-, 6-, and 8-box conditions, SWMBE12 = aggregates errors across trials and used for composite analysis, SWMS = represents the strategy score, VRMFRDS = the number of correctly recalled words during free recall, VRMIRTC = reaction time for correct responses in immediate recognition, and VRMDRTC = reaction time for correct responses in delayed recognition.

## Data Availability

The raw data supporting the conclusions of this article will be made available by the authors, without undue reservation.

## Abbreviations

The following abbreviations are used in this manuscript:

AUS	American University of Sharjah
UAE	United Arab Emirates
25-OHD	25-hydroxyvitamin D
CANTAB	Cambridge Neuropsychological Test Automated Battery
CVD	Cardiovascular Disease
PRM	Pattern Recognition Memory
SWM	Spatial Working Memory
PAL	Paired Associates Learning
OTS	One Touch Stocking of Cambridge
VRM	Verbal Recognition Memory
PFC	Prefrontal Cortex
LTP	Long-term potentiation
WHO	World Health Organization
